# The marginal benefits of healthcare spending in the Netherlands: Estimating cost‐effectiveness thresholds using a translog production function

**DOI:** 10.1002/hec.3946

**Published:** 2019-08-30

**Authors:** Niek Stadhouders, Xander Koolman, Christel van Dijk, Patrick Jeurissen, Eddy Adang

**Affiliations:** ^1^ Scientific Institute for Quality of Healthcare Radboud University Medical Center Nijmegen Netherlands; ^2^ Talma Institute, Department of Health Sciences VU University Amsterdam Amsterdam Netherlands; ^3^ National Health Care Institute Diemen Netherlands; ^4^ Radboud Institute for Health Sciences Radboud University Medical Center Nijmegen Netherlands

**Keywords:** cost‐effectiveness, health care spending, QALY, threshold, translog function

## Abstract

New technologies may displace existing, higher‐value care under a fixed budget. Countries aim to curtail adoption of low‐value technologies, for example, by installing cost‐effectiveness thresholds. Our objective is to estimate the opportunity cost of hospital care to identify a threshold value for the Netherlands. To this aim, we combine claims data, mortality data and quality of life questionnaires from 2012 to 2014 for 11,000 patient groups to obtain quality‐adjusted life‐year (QALY) outcomes and spending. Using a fixed effects translog model, we estimate that a 1% increase in hospital spending on average increases QALY outcomes by 0.2%. This implies a threshold of €73,600 per QALY, with 95% confidence intervals ranging from €53,000 to €94,000 per QALY. The results stipulate that new technologies with incremental cost effectiveness ratios exceeding the Dutch upper reference value of €80,000 may indeed displace more valuable care.

## 1 INTRODUCTION

Medical innovations have been a major driver of health care growth (Smith, Newhouse, & Freeland, 2009) but also brought along major increases in health and longevity (Cutler & McClellan, 2001; Skinner & Staiger, 2015). Recently, however, a more disturbing trend has become visible as pharmaceutical companies target smaller patient groups, and new drugs are becoming much more expensive at modest effectiveness gains (Pearson, 2017). For example, Spinraza®, a new drug targeting the orphan disease spinal muscular atrophy, entered the U.S. market in 2017 with estimated treatment costs of $375,000 to $750,000 per patient per year. More and more countries question whether these new medicines should be reimbursed. Denmark refused reimbursement of Spinraza®, citing unacceptable high costs. In the United Kingdom, reimbursement was refused pending price negotiations. The Irish National Centre for Pharmacoeconomics suggested a tenfold decrease in the price of Spinraza®, estimated at €501,069 per QALY, before reimbursement would be cost‐effective (NCPE, 2017). In the Netherlands, a price reduction of 85% was deemed necessary before uptake into the mandatory benefit package would be advised (ACP, 2018). Under a fixed health budget, new technologies require disinvestment of existing care (Hollingworth et al., 2015). Concerns regarding value‐for‐money of new technologies create an increasingly constrained spending environment (Robinson, 2015).

To guide spending decisions, health losses due to disinvestment should be compared with the gains of innovations. This idea is embodied in cost‐effectiveness thresholds: New technologies should add more value than a predefined threshold in order to be reimbursed (Neumann, Cohen, & Weinstein, 2014). Countries using thresholds include the UK, New Zealand, Australia and Ireland (Edney, Afzali, Cheng, & Karnon, 2018; Eichler, Kong, Gerth, Mavros, & Jönsson, 2004; Harris, 2016; O'Mahony & Coughlan, 2015). The Netherlands currently uses a range of reference values for new drugs of between €20,000 and €80,000 (Reckers‐Droog, van Exel, & Brouwer, 2018). However, these thresholds have no empirical base and thus may not truly reflect the opportunity costs, risking inefficient reimbursement decisions (Thokala, Ochalek, Leech, & Tong, 2018). In general, a distinction is made between demand‐side and supply‐side thresholds, with demand‐side thresholds reflecting willingness‐to‐pay and supply‐side thresholds reflecting opportunity costs of spending decisions (Himani Pandey, Paulden, & McCabe, 2018). For the UK, utilising regional variation in spending and outcomes, a supply‐side threshold of £12,936 per QALY was estimated (Claxton et al., 2015a). Recently, cost‐effectiveness thresholds have also been estimated for Spain, Australia, the United States and Canada (Ariste & Di Matteo, 2017; Edney et al., 2018; Vallejo‐Torres, García‐Lorenzo, & Serrano‐Aguilar, 2017; Woods, Revill, Sculpher, & Claxton, 2016).

This paper applies a novel approach for threshold estimation to hospital care in the Netherlands. We define opportunity costs as the health effect of a marginal change in spending for the average patient group. We restrict our analysis to the hospital sector, as this is where opportunity costs for new drugs and innovations are likely to fall. Other thresholds may apply if expenses are reduced in other sectors (e.g. primary care and tertiary care) to fund new technologies in the hospital sector. QALYs are constructed by combining gains due to lower mortality and gains due to increases in quality of life of all patients (Gheorghe, Brouwer, & van Baal, 2015). We define a production function with spending and the number of patients as inputs and QALYs as outputs for 11,000 patient groups based on gender, disease category and age category. We approximate the hospital production function using a translog specification and estimate a fixed effects model on panel data covering 2012–2014.

Threshold estimations are known to be sensitive to endogeneity (Martin, Rice, & Smith, 2008). This is especially troubling when focusing on spending that aims to reduce mortality as the health care costs involved with the last year of life are known to be substantial (Polder, Barendregt, & van Oers, 2006). Failure to account for these costs could underestimate the effect of health care on survival. As these costs are well studied and known for the Dutch situation (van Baal et al., 2011), we have the opportunity to correct for them. Furthermore, the translog specification accounts for exogeneous changes in health status that may confound the results, as increases in population health are likely to be reflected in reduced patient numbers. As robustness tests for omitted variable bias, we include general health trends (smoking, obesity and alcohol abuse).

Estimation of the translog function renders the marginal effect of spending on the mean patient group, which can be interpreted as a supply‐side cost‐effectiveness threshold (Woods et al., 2016). This may provide information for Dutch policy makers in reimbursement decisions and strengthen the empirical base for using a threshold. Furthermore, we estimate patient group thresholds separately, which may point out inefficiencies in current spending allocation.

## 2 DATA AND METHODS

### 2.1 Data transformations

**Figure 1:**
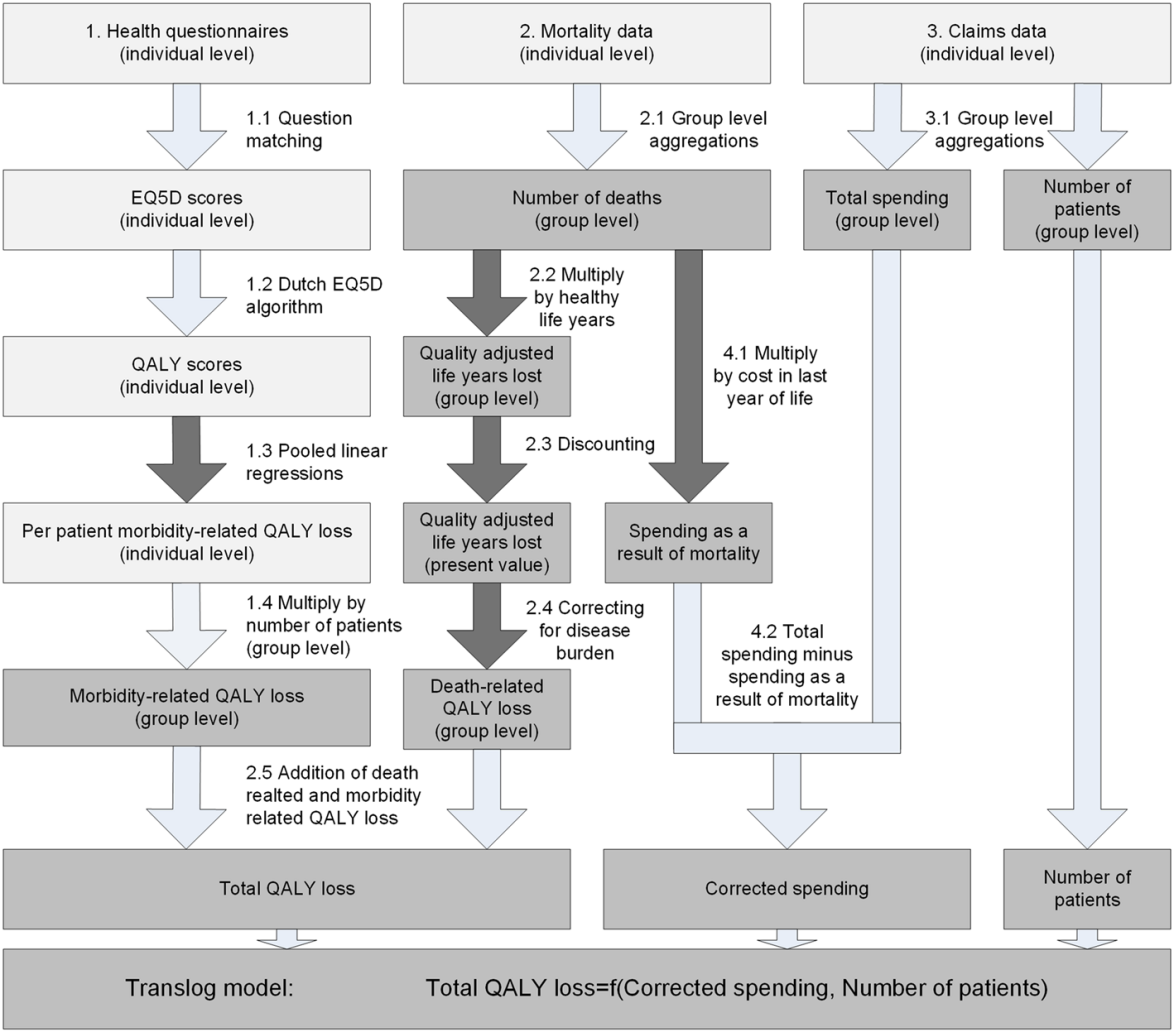
Data transformation and estimation strategy [Colour figure can be viewed at http://wileyonlinelibrary.com]

In the hospital sector, patients lose QALYs as a result of premature deaths (death‐related QALY loss) and lower quality of life while being ill (morbidity‐related QALY loss). Consequently, extra spending may add QALYs resulting both from prevention of premature deaths and increasing the quality of life of patients. In previous research, elasticities of spending on mortality were estimated, after which the outcome was transformed to QALYs (Claxton et al., 2015a). Due to availability of rich data, including health questionnaire outcome data, we were able to first transform both the mortality data and the health questionnaire data to obtain estimated total QALYs (sum of death‐related QALYs and morbidity‐related QALYs) and then estimate the effect of spending on total QALYs. Figure 1 shows the transformations we used to arrive at the level of analysis. Light blocks represent individual level data and dark blocks represent group level data. The arrows show the transformation steps. Transformations that introduce uncertainty (dark arrows) were subject to Monte Carlo analyses.

We combined three datasets on patient group level: health questionnaires, mortality statistics and hospital claims. Hospital claims data contained the euro amount of the claim, a patient follow‐up code, patient gender and age and a DBC code (Dutch alternative to the DRG system) for all hospitals in the Netherlands from 2012 to 2014 (Zorginstituut Nederland, 2017). Claims data prior to 2012 were considered insufficiently comparable due to differences in data collection. Data after 2014 were incomplete at the time of analysis and would introduce selection bias. We defined patient groups based on gender, 5‐year age category and disease group. A classification matrix was used to categorise DBC codes into 405 disease groups, based on 3‐digit codes from the International Classification of Diseases, version 10 (ICD‐10). With two gender groups, 21 age groups and 405 disease groups, 17.010 possible patient group combinations were defined. Of these, 11.079 contained claims. We aggregated claims data to patient group level to obtain total spending per patient group and the number of patients per patient group (Step 3.1). Patients submitting claims in multiple ICD‐groups feature in multiple patient groups. In total, 91% of total hospital spending was attributed to these patient groups. The remainder mainly constituted additional diagnostics and medication that could not be matched to individual DRGs.

### 2.2 From health questionnaires to morbidity‐related QALY loss

Health‐related questionnaires were collected annually from a representative sample of the Dutch population (CBS, 2010–2015). Health status of respondents above 50 years was routinely included in the questionnaires, which allowed us to construct EQ‐(5)D scores. Respondents could be divided into gender‐based 5‐year age groups and whether they visited a hospital during the year.

Morbidity‐related QALY losses on a patient group level were constructed in four steps (1.1 to 1.4 in Figure 1). In Step 1.1, we matched health status questions to a validated QALY‐measurement tool (Gheorghe et al., 2015) to obtain individual EQ‐5D scores. In Step 1.2, using a Dutch EQ‐5D algorithm (Lamers, McDonnell, Stalmeier, Krabbe, & Busschbach, 2006), individual EQ‐(5)D scores were transformed into individual QALY scores. In Step 1.3, following Edney et al. (2018), we estimated changes in patient QALY scores over time, correcting for demographic trends (see Appendix A). Estimations resulted in a time trend in morbidity‐related QALY loss per hospitalised patient by age group and gender. A pooled linear regression estimation rendered mean differences between patients and non‐patients, which could be interpreted as the potential health gains the hospital sector could still achieve (see Appendix A). Combining the two estimates renders per patient group the mean number of QALYs lost due to illness and the mean change in patient QALYs over time. To incorporate uncertainty surrounding the estimates, we included this step in the Monte Carlo uncertainty analysis. In Step 1.4, these outcomes were multiplied by the base number of patients in each patient group in 2012, rendering total patient group morbidity‐related QALY loss.

We assumed that the average QALY scores of patients before they visit the hospital remained constant over time and were not affected by exogenous increases in the health of the population (confounding by indication). If the population would get healthier due to factors outside the health sector, the chance of becoming a patient in a given year may decline. This would reduce the size of the patient group, but mean patient health may be unaffected. Therefore, changes in patient QALY scores could be fully attributed to the health sector. Extra spending may increase quality of life of patients who would not have died but may also avert deaths of patients who would have, rendering the effect on average quality of life of all patients ambiguous (Ochalek et al., 2015). Although the health questionnaires were used to measure the primary effect of increases in quality of life of patients (Step 1.1 to 1.4), the effect of lower mean quality of life due to increases in survival was introduced in Step 2.3 (described below).

### 2.3 From mortality data to death‐related QALY loss

Mortality statistics were collected by Statistics Netherlands and contained all nationwide deaths in a given year including information on age, gender and primary cause of death according to the 3‐digit ICD‐10 codes. The ICD‐10 codes allowed appointment of deaths to the same patient groups as defined by claims data (Step 2.1). In 3713 patient groups, at least one death was recorded. In total, 94% of all deaths were appointed to a patient group with positive spending.

To transform the number of deaths to death‐related QALY loss per patient group, we followed Claxton et al. (2015a). Contrary to the UK, estimates of healthy life expectancy were readily available in the Netherlands (CBS, 2018). This allowed us to compute healthy years of life lost for deaths in all age groups (Step 2.2). Some of the benefits of averted deaths are in the future, requiring discounting to calculate the net actuarial benefit of averted deaths. Following Dutch guidelines, in Step 2.3, we apply a discount rate of 1.5% (Zorginstituut Nederland, 2015). As literature provides no consensus on the appropriate discount rate (Claxton, Paulden, Gravelle, Brouwer, & Culyer, 2011), we allowed discount rates to vary between 0 and 5% in our sensitivity analysis. If a death is averted, a patient may not fully return to the average health status of the population. Therefore, in Step 2.4, we used Dutch disease‐specific disability‐adjusted life years (DALY) estimates from Hoeymans et al. (2014) to correct for burden of disease (see Table 2). DALY values ranged between 0 and 1 and included utility losses due to premature death and lower health in life (Hoeymans et al., 2014). In our research, healthy life years were reduced by the relative DALY burden (Step 2.4), for example, a DALY value of 0.1 resulted in a 10% reduction in disease specific healthy life years relative to the healthy population. Steps 2.2 to 2.4, rendering the number of QALYs lost due to mortality (Gafni & Birch, 1993), introduced extra uncertainty in the estimates, which was evaluated using Monte Carlo analysis. By adding the number of QALYs lost due to mortality to the number of QALYs lost due to morbidity (Step 2.5), total QALY loss for each of the 11,000 patient groups was obtained.

### 2.4 Correcting for reverse causality

Reductions in mortality may lower health spending due to fewer mortality‐related costs, and increased spending can reduce mortality. Due to this reverse causality, straightforward estimation would result in underestimation of the true effect of extra spending on outcomes, that is, an upward biased threshold. Because last year of life costs are known in the Netherlands, we were able to correct for the cost resulting from changes in mortality directly and isolate the effect of changes in spending on changes in mortality. Although a strong and valid instrument is preferred to correct for endogeneity, direct correction may be a good alternative when no valid IVs are at hand (Moreno‐Serra & Smith, 2015). To this aim, we split the bidirectional causality by disaggregating spending (*S*_*i*_) into last year of life costs (*LYoL*_*i*_) that resulted from mortality and the costs that do not result from mortality, which we call corrected spending (*C*_*i*_):

(1) $$S_{i}={\text{LYoL}}_{i}+C_{i}$$

By construction, exogenous changes in mortality only influence *LYoL*_*i*_, allowing estimation of the effect of changes in corrected spending (*C*_*i*_) on mortality. In order for Equation (1) to hold, *LYoL*_*i*_ should be independent of changes in mortality.

(2) $$\mathrm{Cov}\;\left({\text{LYoL}}_{i},{\text{mortality}}_{i}\right)\equiv 0$$

If lower mortality changed the average LYoL‐costs, for example, if predominantly high‐cost deaths were averted, the estimate would be biased downward, whereas if mostly low‐cost deaths were averted, the effect would be biased upward. Moreover, this correction may be incomplete: If LYoL‐costs would increase over time, Equation (1) insufficiently corrects for reverse causality, biasing the estimated thresholds upwards.

For the Netherlands, mean LYoL‐costs were known for age groups and gender (van Baal et al., 2011). In Step 4.1, we multiplied the LYoL‐costs by the number of deaths for each of the 11,000 patient groups to obtain the total amount of spending as a result of mortality (*LYoL*_*i*_). Due to the uncertainty surrounding LYoL‐costs, Monte Carlo uncertainty analysis was used. In Step 4.2, we subtracted the LYoL‐costs from total spending to obtain corrected spending (*C*_*i*_).

### 2.5 Correcting for omitted variable bias

Underlying health status may influence both spending and health outcomes: If fewer patients get ill and die from a disease, for example, due to healthier lifestyle, costs for the patient group may be lower and fewer QALYs are lost due to both mortality and morbidity. Straightforward estimation would erroneously attribute the health gains to the hospital sector, causing thresholds to be biased downward. To correct for omitted variable bias (OVB), others have attributed a fixed part of the gains to factors outside the health sector (Cutler & McClellan, 2001; Hall & Jones, 2004). We correct for OVB by using changes in the number of patients as proxy for health trends. The underlying assumption is that when health of a patient group improves, the number of patients that visit the hospital decreases. This assumption is violated if health trends change treatment intensity and outcomes while keeping patient numbers stable, which could happen in the case of waiting lists. However, waiting lists in the Netherlands only exist for a small number of patient groups and are relatively low (Siciliani, Moran, & Borowitz, 2014). When hospitals respond to lower patient numbers by attracting new, healthier patients through supplier‐induced demand, OVB may also remain. Other potential sources of endogeneity include time effects. Cost and outcome variables may be correlated to previous years' values. We corrected for this using a fixed effects model (Wooldridge, 2010). Furthermore, health shocks may affect future spending, which may bias the estimators upwards. As robustness checks, we corrected for health shocks by including time dummies and lagged effects.

### 2.6 Empirical strategy

We used total QALY loss, corrected spending and the number of patients for each of the 11,000 patient groups as inputs for our empirical estimation strategy. For each patient group *i*, we define QALYs (*Q*) as an unknown function of corrected spending (*C*) and number of treated patients (*N*):

(3) $$Q_{i}=f\left(C_{i},N_{i}\right).$$

We assumed diminishing marginal returns: *f* ′ (*C*_*i*_) < 0,*f* ′  ′ (*C*_*i*_) > 0, and assumed that the production function was differentiable at relevant intervals (Boisverf, 1982). We approach the unknown function *f*(*c*_*i*_) at the mean by defining the second order Taylor polynomial:

(4) $$\log\left(Q_{\mathrm{it}}\right)=\alpha_{1}+\alpha_{2}T_{\mathrm{it}}+\beta_{1}\mathrm{Log}\left(C_{\mathrm{it}}\right)+\frac{1}{2}\beta_{2}\mathrm{Log}{\left(C_{\mathrm{it}}\right)}^{2}+\theta_{1}\mathrm{Log}\left(N_{\mathrm{it}}\right)+\frac{1}{2}\theta_{2}\mathrm{Log}{\left(N_{\mathrm{it}}\right)}^{2}+\;\;\beta_{3}\mathrm{Log}\left(C_{\mathrm{it}}\right)\mathrm{Log}\left(N_{\mathrm{it}}\right)+\varepsilon_{i}+\epsilon_{\mathrm{it}}$$

Where *α* is the group specific productivity parameter, *T* is the trend in time *t*, the *β* coefficients are the cost elasticity parameters and the *θ* coefficients are the treatment elasticity parameters. *ε*_*i*_ contains fixed effects, and *ϵ*_*it*_ is the error term. Evaluated at the mean, the translog function approximates the unknown production function. Using a translog function to estimate the marginal effect of spending is preferred over commonly used explicit specifications—such as the linear or Cobb‐Douglas model—if the elasticity is nonlinear and the elasticity of substitution is unknown (Boisverf, 1982; Pavelescu, 2011), which are both likely for the heterogeneous patient groups.

The elasticity of spending *e* for the mean patient group was obtained by the first derivative of log(*Q*_*it*_) with respect to log (*C*_*it*_):

(5) $$e=\frac{\delta \log\left(Q_{i,t}\right)}{\delta \log\left(C_{i,t}\right)}=\beta_{1}+\beta_{2}\mathrm{Log}\left({\bar{C}}_{i,t}\right)+\beta_{3}\mathrm{Log}\left({\bar{N}}_{i,t}\right)$$

Next, the elasticity was evaluated at the mean to obtain the marginal effect of spending for the mean patient group:

(6) $$\frac{\delta \left(C_{i,t}\right)}{\delta \left(Q_{i,t}\right)}=\frac{{\bar{C}}_{i,t}}{e*{\bar{Q}}_{i,t}}$$

Uncertainty with respect to the construction of the outcome variable was incorporated into the estimation by running 10,000 Monte Carlo simulations for all transformations combined and separately for each individual transformation (Claxton, 2008). We incorporated uncertainty regarding the values for healthy life expectancy, quality of life gains, burden of disease and cost in last year of life (see Section 2.1).

### 2.7 Robustness checks

As robustness check, we tested differences in elasticity with respect to gender, age category and main disease category (Appendix B). However, these estimates should be treated with caution, as digression from the population mean reduces the accuracy and the validity of the Taylor approximation (Boisverf, 1982). As marginal values may depend on outcome variable and model specification used (Gallet & Doucouliagos, 2017), alternative outcome variables and model specifications were explored (Appendix C). We separately estimated mortality, death‐related QALY loss and morbidity‐related QALY loss as outcome measures. Furthermore, we estimated alternative model specifications, linear models and Cobb‐Douglas (per patient) specifications. We included health trends and health shocks, specifically the percentage of (heavy) smokers, the percentage of obesity and the percentage of heavy drinkers.

As patients could have been part of more than one patient group in the case of multimorbidity, in theory, spending on one disease‐specific patient group may influence mortality of another. We corrected for multimorbidity by defining unique patient groups based on primary diagnosis. After appointing deaths to the unique patient groups based on spending patterns on secondary diagnoses (proportionally or through OLS estimation), data transformations and estimations were performed according to Figure 1.

## 3 RESULTS

### 3.1 Summary statistics

Summary statistics of the data are presented in Tables 1, 2, 3. Real hospital spending was relatively stable around €21 billion between 2012 and 2014. The number of patients declined slightly from 7.4 million to 7.1 million, as did the total number of deaths from 141,000 to 139,000. Per patient hospital spending was highest between the ages of 76 and 80 and thereafter declined. Spending and mortality was highest for cancer and circulatory diseases, whereas most patients visited the hospital with diseases related to the eye and ear and external causes and injuries.

**Table 1:** Summary statistics over time

Aggregate statistics	2012	2013	2014
*Total hospital spending in millions of 2014 Euro*	20,757	21,031	20,702
*Number of unique patients (x1000)*	7,379	7,184	7,112
*Total deaths (x1000)*	141	141	139

**Table 2:** Summary statistics by age group in 2014

Age group	Hospital spending (million euro)	Number of patients	Number of deaths*	Healthy life expectancy (net present value)	Costs in last year of life
0 years	651	162,972	630	38.2	€ 25,219
1 to 5 years	444	283,356	99	37.5	€ 8,225
6 to 10 years	353	271,087	75	35.7	€ 7,405
11 to 15 years	426	275,794	100	33.9	€ 8,380
16 to 20 years	444	286,219	226	31.9	€ 10,248
21 to 25 years	529	315,197	266	29.8	€ 12,626
26 to 30 years	731	350,413	364	27.7	€ 15,511
31 to 35 years	799	361,227	444	25.5	€ 17,409
36 to 40 years	785	354,774	614	23.1	€ 18,997
41 to 45 years	1,028	442,103	1,232	20.5	€ 22,535
46 to 50 years	1,268	492,095	2,152	18.0	€ 28,168
51 to 55 years	1,528	531,656	3,647	15.5	€ 33,159
56 to 60 years	1,749	537,316	5,336	13.1	€ 35,690
61 to 65 years	2,031	567,446	7,880	10.9	€ 34,355
66 to 70 years	2,334	589,323	11,729	8.7	€ 31,097
71 to 75 years	1,975	458,821	13,285	6.5	€ 28,362
76 to 80 years	1,689	374,030	17,286	4.6	€ 24,053
81 to 85 years	1,188	267,380	23,307	3.4	€ 17,086
86 to 90 years	562	138,191	24,875	2.4	€ 10,969
91 to 95 years	172	45,263	18,699	1.6	€ 6,607
95+ years	24	6,883	6,977	1.2	€ 4,249

* Due to privacy issues, the total number of deaths is based on official statistics, which use different age groups (1 to 4 years, 5 to 9 years, etc.).

**Table 3:** Summary statistics by disease category in 2014

Disease group	LYoL corrected spending (million euro)	Number of treated patients	Number of deaths	Burden of disease men (women)
Certain infectious and parasitic diseases	€ 210	120,971	3,104	0.051 (0.045)
Neoplasms	€ 3,655	1,284,510	44,808	0.225 (0.246)
Diseases of the blood and blood‐forming organs and certain disorders involving the immune mechanism	€ 138	73,034	485	0.172 (0.166)
Endocrine, nutritional and metabolic diseases	€ 436	348,113	3,529	0.198 (0.198)
Mental, behavioural and neurodevelopmental disorders	€ 140	161,118	10,193	0.385 (0.394)
Diseases of the nervous system	€ 667	568,905	6,849	0.162 (0.089)
Diseases of the eye and adnexa and diseases of the ear and mastoid process	€ 921	1,937,672		0.123 (0.123)
Diseases of the circulatory system	€ 3,051	1,432,255	37,862	0.29 (0.288)
Diseases of the respiratory system	€ 992	647,769	10,454	0.181 (0.166)
Diseases of the digestive system	€ 1,272	622,379	4,374	0.172* (0.166*)
Diseases of the skin and subcutaneous tissue	€ 278	528,068	309	0.07 (0.07)
Diseases of the musculoskeletal system and connective tissue	€ 2,050	1,375,562	1,046	0.159 (0.137)
Diseases of the genitourinary system	€ 1,450	873,525	3,076	0.142 (0.142)
Pregnancy, childbirth and the puerperium and certain conditions originating in the perinatal period	€ 597	292,207	384	0.172* (0.166*)
Congenital malformations, deformations and chromosomal abnormalities	€ 180	108,750	430	0.132 (0.132)
Symptoms, signs and abnormal clinical and laboratory findings, not elsewhere classified (nec)	€ 747	983,587	5,507	0.172* (0.166*)
External causes of morbidity and injury, poisoning and certain other consequences of external causes	€ 2,931	2,164,496	6,813	0.172* (0.166*)

* The mean BoD is used when no disease specific BoD is available.

### 3.2 Main specification results

Table 4 shows the results of the fixed effects specification. The spending coefficients are jointly significant (*p* < .01). Evaluating the coefficients at the mean [(ln( $\bar{C}$); ln( $\bar{N}$))=(11.97; 5.10)] resulted in a mean elasticity of spending of −0,156 1Note the negative sign on the elasticity, signaling that an increase in spending on a patient group reduces the number of QALYs lost due to mortality and illness. [Equation (5)]. The elasticity turned more negative the more patients per patient group and less negative when spending per patient group was higher, indicating complementarity of inputs and diminishing marginal returns. Next, we translated the mean elasticity of spending to a marginal effect at the arithmetic mean according to Equation (6): [F( $\bar{Q}$, $\;\bar{C}$) =(145.73, €1,678,091). A 1% increase in spending (€16.781) on the mean patient group was associated with a reduction in QALY loss of 0.156%*145.73 = 0.23 QALYs, resulting in a threshold of € 73,626. Bootstrapping 100 repetitions, assuming normal deviation around the mean, we found a 95% confidence interval around the threshold value between € 59,178 and €88,076. The standard error around the threshold was €7,372.

**Table 4:** Main regression results

*N* = 13,618	F(6,13611) = 170.35		Prob > F = 0.000		R^2^ = 0.0817
Variable	Coefficient	Standard error	t‐value	p‐value	95% confidence interval
Ln (spending)	−0.0461	0.0779	−0.59	0.554	[−0.1988;0.1066]
Ln (spending)^2^	0.0033	0.0060	0.55	0.583	[−0.0085; 0.0151]
Ln (spending)*Ln (patients)	−0.0372	0.0127	−2.93	0.003	[−0.0620;‐0.0123]
Ln (patients)	1.3012	0.0837	15.55	0.000	[1.1372; 1.4653]
Ln (patients)^2^	0.0222	0.0075	2.95	0.003	[0.0074; 0.0370]
Time trend	−0.0185	0.0017	−10.89	0.000	[−0.0219; ‐0.0152]
Constant	35.330	3.456	10.22	0.000	[28.556;42.105]

Data transformations increase uncertainty, which is not incorporated into the thresholds. Therefore, we used Monte Carlo simulations (Table 5). Including transformation uncertainty increased our confidence intervals to between €54,000 and €94,000. Most uncertainty is attributable to LYoL‐costs.

**Table 5:** Outcomes Monte Carlo distributions

Variable	Distribution	Range	Source	Standard error	Confidence interval (Euro per QALY)
Healthy life expectancy	Normally	SD from source	Central Bureau of Statistics (CBS) Netherlands	€ 877	€ 69,400 ‐ € 75,800
Quality of life gains	Normally	SD from regressions	CBS health questionnaires	€ 732	€ 69,700 ‐ € 75,100
Burden of disease	Continuous	Between BoD‐value and 1	Volksgezondheid en Zorg (2011)	€ 517	€ 66,800 ‐ € 70,500
Cost in last year of life	Normally	SD is assumed to be 10% of mean	Van Baal et al. (2011)	€ 2,767	€ 59,900 ‐ € 80,800
All variables				€ 2,789	€ 56,600 ‐ € 79,000
Total uncertainty including uncertainty of the main specification				€10,161	€54,000 ‐ €94,000

Abbreviation: BoD: burden of disease.

In line with standard Dutch guidelines on HTA research, we used a discount rate of 1.5% to discount future health gains (Zorginstituut Nederland, 2015). As most gains of deaths averted are in the future, a higher discount rate increases the threshold. For example, our point estimate for a discount rate of 3% was €80,800, and a discount rate of 5% resulted in a threshold of €90,200. A 0% discount rate, which has also been advocated in the literature (Parsonage & Neuburger, 1992), lowered the threshold to €66,500. Each percent increase in the discount rate shifted the threshold upwards by €4,700.

### 3.3 Results per patient group

We estimated separate thresholds per gender, age and disease groups (see Appendix B). A higher threshold was found for males but not significantly. This may indicate that spending on females is more beneficial, possibly due to a higher life expectancy. Thresholds over age groups are strikingly constant save the neonates and 95+ year‐olds, indicating that spending on age groups largely takes into account healthy life years to be gained and that a discount rate of 1.5% seems appropriate.

Differences between ICD‐10 categories are larger. For some categories, high thresholds were found, that is, diseases of the blood and pregnancy, whereas lower thresholds were found in other categories, specifically diseases of the nervous system and diseases of the skin. Potential explanations for these differences include inefficient allocation patterns, differences in QALY valuations, deviations from the mean in the translog estimation and measurement errors. More research is required to assess the clinical relevance of these differences. For most patient groups valid and significant thresholds were found, indicating the robustness of the estimation strategy.

### 3.4 Robustness checks

The robustness checks show that the outcome was sensitive to the structural model employed, but the translog model generally was robust to different specifications (see Appendix C). When only patient groups with mortality were included a value of €61,100 per QALY was found, suggesting that our combined measure of disease and mortality related QALYs may not fully capture all health gains. Excluding morbidity‐related QALY loss (Steps 1.1–1.4) raised the threshold to €89,000 per QALY. Estimating the relation between spending and mortality directly resulted in an estimated effect of €275,000 per death averted. Backwards calculations to QALYs rendered a threshold of €42,000 per QALY. Using 2‐year QALY gains as outcome measure lowered the threshold slightly to €60,000 per QALY, suggesting spending affecting health outcomes primarily in the same year but possibly also in the next years. However, estimating the effect of spending in year *t* on outcomes in year *t*+1 rendered insignificant and economically unlikely results. This may indicate that our 3‐year panel dataset is too limited to estimate robust lagged effects. Neither time dummies to correct for technology shocks nor health trends to correct for omitted variable bias influenced the threshold estimates, suggesting our OVB correction is appropriate.

Multimorbidity corrections produced divergent results. Proportional multimorbidity corrections resulted in a threshold of €201,000 per QALY (€ 143,000–€ 271,000 per QALY). However, a proportional distribution of deaths implies that higher spending increases the proportion of total deaths being appointed to that patient group. This increases reverse causality, biasing the threshold upwards. When patient group mortality was estimated using OLS, we obtained a threshold of €49,600 per QALY (€ 46,000–€ 63,000 per QALY). However, estimating the number of deaths based on spending patterns aggravates truncation bias, as negative estimates are not allowed. This may bias the threshold downwards. Although multimorbidity corrections are promising, additional corrections may be required to alleviate bias.

## 4 DISCUSSION

This paper presents a novel method to estimate the opportunity costs of care by combining mortality related outcomes and quality of life of patients into one outcome measure and relating it to changes in spending over time. To this aim, we built upon panel data methodology (Felder, 2006; Hall & Jones, 2004) and QALY estimation methods (Claxton et al., 2015a; Gheorghe et al., 2015), accounting for some of the issues raised in QALY threshold estimation (Barnsley, Towse, Karlsberg Schaffer, & Sussex, 2013; Raftery, 2014). Results indicate that in the Netherlands, at the margin, a QALY costs between €53.000 and €94.000 to produce, with a point estimate of €73.600. Standard economic theory suggests that under a fixed budget, new technologies would need to have an incremental cost effectiveness ratio of below €73.600 per QALY in order to increase population health. Although the analysis is rather inclusive, much uncertainty remains. We discuss how the results relate to earlier findings, the risks of bias of the estimates, the factors that could explain uncertainty and next steps to improve the estimates.

### 4.1 Relation to the literature

Several studies have estimated thresholds for high‐income countries, ranging from €18,000 to €200,000 per QALY (Claxton et al., 2015b; Thokala et al., 2018; Vallejo‐Torres et al., 2016; Vallejo‐Torres et al., 2017). Literature suggests that willingness‐to‐pay estimates tend to be higher than supply‐side estimates (Vallejo‐Torres et al., 2016). Our supply‐side estimates are broadly in the same range as Dutch willingness‐to‐pay estimates for hospital care of between €13,000 and €110,000 per QALY (Bobinac, Van Exel, Rutten, & Brouwer, 2010; Bobinac, van Exel, Rutten, & Brouwer, 2014; Nimdet, Chaiyakunapruk, Vichansavakul, & Ngorsuraches, 2015).

Research from the US finds marginal costs to save a life at age 60–64 of around $800,000 2At 10.9 healthy life years (Table 2), and 2014 dollar exchange rates this would accrue to €69,242 per QALY. (Hall & Jones, 2004). For Switzerland, a marginal cost to save a life is found between 700,000 Franc to 3.5 million Franc 3This would be between €55,107 and €275,535 per QALY in 2014. (Felder, 2006). These results are consistent with our estimates. Other research finds significantly higher marginal values, that is, lower thresholds. For example, cross‐country research from 2013 finds marginal effects of €20,000 to €30,000 per life year gained for the Netherlands 4A ratio of ~0.6 QALY per life year would imply thresholds of € 37,785 ‐€ 56,700 per QALY in 2014 euros (Heijink, Koolman, & Westert, 2013). One of the most extensive lines of research up to date has been performed by the Centre of Health Economics in England. This line of research, utilising regional variation in spending, finds threshold values for England of £13,000 per QALY 5Or €19,200 in 2014 Euros (Claxton et al., 2015a; Drummond, Sculpher, Claxton, Stoddart, & Torrance, 2015). Extrapolating this finding to the Netherlands, accounting for income elasticity, renders threshold values of € 21,000 to € 29,000 (Woods et al., 2016). Possibly, the UK is more efficient or uses cost‐effectiveness thresholds more strictly than the Netherlands. Also, thresholds may increase over time, as diminishing marginal returns make it more and more difficult to increase population health by one QALY (Barro, 1996; Murphy & Topel, 2003). Research from 2001 shows that the marginal cost per life year gained for a 65‐year old increased from $121,000 in 1985 to $141,000 6This is 203,724.08 US Dollars of 2014, or 153,176 Euros of 2014 per life year saved. Using a QALY per year ratio of ~0.6 this would be €255,293 per QALY. In comparison, we find for this age category a marginal value of € 65,000 per QALY. in 1995 (Cutler & McClellan, 2001).

### 4.2 Factors influencing threshold estimation

We distinguish between factors increasing uncertainty and factors that could potentially bias the estimators. Claims data may not represent unit costs due to internal cost shifting between departments of the hospital, which may increase uncertainty. The mortality dataset could contain measurement and classification errors. For example, in 2013 primary cause of death classification was altered (Harteloh, 2014). This may increase uncertainty of our estimations, but time dummies do not indicate any bias. Questionnaires used to infer quality of life might contain sampling uncertainty, interrater variation and framing issues, amongst others. For example, very ill patients may be underrepresented. Although quality of sampling by the Dutch Statistical Bureau was excellent, some inaccuracies may be expected. These data limitations primarily increase model uncertainty but may also lead to small sampling bias in unknown direction.

Increases in model uncertainty by data transformations are captured by the Monte Carlo simulations. However, some extrapolations were necessary, requiring additional assumptions. For example, we had to assume that disease‐specific DALYs were stable over age, which may not be valid. Differences in estimations between age groups could in part reflect differences in burden of disease. Morbidity‐related QALY loss for ages under 50 was extrapolated, assuming smooth trends. However, nonlinear trends may be present, for example, when the very young patients are healthier than the linear extrapolation predicts. This could bias the marginal effect downwards. In addition, morbidity‐related QALY loss is not disaggregated to disease category, which may explain differences in disease category thresholds. The approximation of the EQ‐5D by the health questionnaires was not validated and may be imprecise. Also, uncertainty in translation of the EQ‐5D to QALY values was not incorporated. The use of QALY‐values from the literature assumes comparability, which may be a strong assumption (Gafni & Birch, 1993). Furthermore, we assume that the change in morbidity‐related QALY loss is constant over time and spending‐related health shocks may be present.

Our indicator may not capture additional health system outcomes, biasing the threshold upwards (Nixon & Ulmann, 2006). For example, in fertility treatments, reductions in morbidity‐related QALY loss and death‐related QALY loss may not fully capture all benefits. In these instances, our estimation underestimates the true benefits of health spending. Furthermore, our data do not incorporate all health spending, such as private spending, spending on primary care and municipal health spending. This could bias the marginal effect upward if these types of spending are complementary to hospital spending. However, research suggests no correlation between spending types (de Jong et al., 2016). Importantly, effects of spending on future mortality and future gains in patient quality of life are not taken into account. This would require additional data years and multiple lag estimation. When multiple lags are tested, LYoL‐costs need to be adjusted for the additional spending in additional years before death (Howdon & Rice, 2018).

Using a direct approach to correct for endogeneity as opposed to IV‐estimation poses the risk of incomplete or imprecise corrections, which would lead to biased estimators. One major factor influencing our estimation is the correction for reverse causality using cost in the last year of life. This is not disaggregated to disease category, which may explain differences in disease categories. For example, cost in last year of life of a patient that died due to external causes (e.g. traffic injuries) may be lower than the cost in the last year of life of a cancer patient. This would have little effect on the main estimation, as the translog function is estimating the elasticity for the mean patient group, which by definition also has mean LYoL‐costs. However, the marginal effect of specific disease groups may be biased upwards or downwards. Secondly, we disregard the possibility that when patients die at the beginning of the year, not all costs in the last year of life fall into the same year. In stable demographic conditions, this effect can be disregarded, but when mortality is decreasing, this would overcorrect for reverse causality, biasing the marginal effect upwards. Lastly, correcting for the costs in the last year of life could cause censoring bias, as patient groups with negative spending (patient groups with low spending, high mortality and lower‐than‐average LYoL‐costs) cannot be log‐transformed. In our analysis, this occurs in less than 1% of patient groups. Nevertheless, future research should take this into account using data sampling and correction methods (Greene, 2005).

In summary, analysis of potential biases is inconclusive on whether the model is overestimating or underestimating the true effect. That not all benefits are included fully and that correction for reverse causality may be incomplete may suggest a downward bias of the estimated elasticity (Raftery, 2014). For example, when only mortality‐related patient groups are included, a larger marginal effect and lower threshold is obtained. Based on this, our estimates may be interpreted as a conservative threshold.

### 4.3 Policy relevance and future recommendations

The findings, as presented in this paper, are important for policy makers in a number of ways. First of all, the results can be used by Dutch policy makers as a reference value to evaluate new technologies. Our estimates are close to the upper bound of the reference value for new pharmaceuticals of €80,000 per QALY. According to our estimates, under a constrained budget, new technologies may displace care valued at €73,600 per QALY, suggesting that new treatments and medicines should provide value of more than €73,600 per QALY to maximise total health. In resource allocation, policy makers should compare hospital spending with the value of all other spending alternatives, not just in healthcare but also to other public spending categories like education or infrastructure. Our methodology is suited to estimate marginal value in other areas of health, but estimating value of other government spending would require different methodologies. Between‐country differences of marginal benefits for different disease categories or age groups could reflect clinical differences between countries. Although our research provides disease‐specific thresholds, several issues require further research before these comparisons may be used to improve allocation of hospital funds. Three additions would be most valuable. Firstly, LYoL costs should be specified to disease category because this is the most important source of transformation uncertainty. Secondly, additional years should be analysed. This serves three goals: to increase the precision around the estimators; to estimate how thresholds change over time; and to reduce the risk of overfitting. Thirdly, QoL monitoring should be improved, for example, by using patient reported outcome measures. These three improvements would allow identification of areas that are relatively overfunded or underfunded with more certainty. The relevance of this research for policy making calls for further studies focusing on a single outcome or disease type—requiring fewer assumptions. Recently, for example, a cost‐effectiveness threshold of €41,000 was estimated for cardiovascular hospital spending in the Netherlands in 2010 (van Baal et al., 2018). This indicates that up to 2010, cardiovascular care may have been relatively cost‐effective, and shifting additional resources to this patient group could have improved total health. To improve efficient allocation of spending, more research on cost effectiveness of single disease groups should be encouraged.

Lastly, other relevant factors need to be taken into account when using thresholds for evaluation of new technologies. Firstly, in our research, QALYs do not include differential preferences regarding burden of disease. It could be that a QALY gain for a patient with high disease burden is valued differently than a QALY gain of any other patient. This would still require policy discretion (Harris, 2016). In addition, for technologies with a non‐marginal budget impact, the thresholds may underestimate the true opportunity costs (Lomas, Claxton, Martin, & Soares, 2018; Paulden, 2016).

To conclude, we set out to use a new and extensive method to calculate the marginal benefits of spending. Application to Dutch hospital data produced a marginal value of €73,600 per QALY, close to the Dutch upper policy reference value of €80,000 per QALY for new technologies. The research shows that, although uncertainty remains, the methodology produces policy makers with informative decision input for resource allocation and new technology assessment. Therefore, it would be valuable to extend, improve and compare the results over more years and different settings.

## Supporting information


**Appendix A:** Estimating gains in quality of life
**Appendix B:** Estimating thresholds per patient category
**Appendix C:** Robustness checks
