# Supplementary material for: The marginal benefits of healthcare spending in the Netherlands: Estimating cost‐effectiveness thresholds using a translog production function
Source: Health Econ. 2019 Aug 30;28(11):1331–44. doi: 10.1002/hec.3946 (PMC6851736; doi:10.1002/hec.3946)
Supplement: Supplementary file 1 — Appendix A: Estimating gains in quality of life Appendix B: Estimating thresholds per patient category Appendix C: Robustness checks [file HEC-28-1331-s001.docx]

**The marginal benefits of healthcare spending in the Netherlands**

**Estimating cost-effectiveness thresholds using a translog production function**

**Appendix 1: Estimating gains in quality of life**

We estimate differences between patient QALYs and non-patient QALYs using pooled OLS with robust standard errors:

1. ${Qaly}_{i}=\beta_{0}+ \boldsymbol{\beta}_{\boldsymbol{x}}{Patient}_{i}\#{gender}_{i}+\beta_{2}{year}_{i}+\beta_{2}{age group}_{i}+\beta_{3}{demographics}_{i}+e_{i}$

Where subscript i refers to respondents. Patient is a dummy variable, which is one if a respondent visited a hospital and zero otherwise. The # denotes an interaction matrix, which obtains beta coefficientsfor each vector. $\boldsymbol{\beta}_{\boldsymbol{x}}$ estimates the difference in QALYs between patients and the healthy population for males and females, respectively. A negative $\boldsymbol{\beta}_{\boldsymbol{x}}$ indicates that patients have lower health than the population, which is what the health sector in theory could achieve if all patients are fully healed. If $\boldsymbol{\beta}_{\boldsymbol{x}}$ is zero, the health sector brings all patients back to the health level of the healthy population.

However, not all QALY gains in the population may be attributed to the health sector. Other factors, such as healthier lifestyles and environments may improve QALYs exogenously. Therefore, we assume that all *patient* QALY gains can be attributed to the health sector due to confounding by indication. If the health level of the patient population rises exogenously, the healthiest patients may not visit the hospital anymore and move to the group of non-patients. This can be corrected for by assuming that the QALY scores of patients before they visit the hospital is not affected by exogenous increases in the health of the population. As a result, exogenous QALY gains are not reflected in patient group QALY levels, and changes in patient QALY scores may be fully attributed to the health sector. To estimate trends in patient QALY score, we use a second pooled OLS estimation:

1. ${Qaly}_{p}=\gamma_{0}+\boldsymbol{\gamma}_{\boldsymbol{x}} {age group}_{p}\#{gender}_{p}\#{year}_{p}+\gamma_{2}{demographics}_{p}+\varepsilon_{p}$

Where subscript p to a subset of respondents that visited the hospital in a given year (patients). The matrix of coefficients $\boldsymbol{\gamma}_{\boldsymbol{x}}$ consists of estimates of annual changes in QALYs for patient groups based on age and gender. A linear extrapolation is used to obtain values for patient groups under 50. The data do not allow disaggregation into disease groups. QALY losses due to morbidity are constructed by subtracting $\boldsymbol{\gamma}_{\boldsymbol{x}}*\mathrm{year}$from $\boldsymbol{\beta}_{\boldsymbol{x}}$ for every patient per patient group. The QALY estimates are extrapolated to patient groups to obtain the total number of QALYs lost to illness per patient group:

$${QALY}_{i,t}^{D}=(\beta_{i}-\gamma_{i}*t)*N_{i,t}$$

Tables 6-7 show the results of the regression on the questionnaires database. The regressions also estimate that on average a male resp. female patient that visited the hospital has 0.08 (se=0.005) and 0.10 (se=0.008) fewer QALYs than the population that did not visit the hospital, corrected for age, demography, time and gender. Females have 0.03 lower QALY rating on average, while all QALY ratings improve over time by 0.005. The number of persons in an age group does not have a significant effect on the QALY rating.

Estimated annual health gain of patients is between 0.0014 and 0.0016 (se=0.0024) QALYs per year, depending on gender and age (table 7).These estimates are not significant and contain a high degree of uncertainty. This reflects the low sample size and small effect size. We incorporate this uncertainty in the Monte Carlo analysis. The results of these regressions are extrapolated to lower age categories using linear extrapolation (figure 4).

| Table 6: health questionnaire QALY scores regression | | | | | |
| --- | --- | --- | --- | --- | --- |
| N=13,618 | F(6,13611) = 170.35 | | Prob>F=0.000 | | R^2^=0.0817 |
| variable | Coefficient | Standard error | t-value | p-value | 95% confidence interval |
| Gender | -0.02936 | 0.00508 | -5.78 | 0.000 | [-0.0393;-0.0194] |
| Patient | -0.08016 | 0.00509 | -15.74 | 0.000 | [-0.0901;-0.0702] |
| Patient x Gender | -0.01870 | 0.07553 | -2.48 | 0.013 | [-0.0335;-0.0039] |
| Time trend | 0.00455 | 0.00146 | 3.11 | 0.002 | [0.0017;0.0074] |
| Age | -0.01332 | 0.00481 | -2.77 | 0.006 | [-0.0227;-0.0039] |
| Demographics | 5.86*10^-8^ | 5.84*10^-8^ | 1.00 | 0.315 | [-5.6*10^-8^; 1.7*10^-7^ |
| Constant | -8.09640 | 2.91358 | -2.78 | 0.005 | [-13.807;-2.385] |

| Table 7: Patient QALY scores time trend regression | | | | | | |
| --- | --- | --- | --- | --- | --- | --- |
| N=8,071 | | F(20,8071) = . | | Prob>F=. | | R^2^=0.0461 |
| variable |  | Coefficient | Robust standard error | t-value | p-value | 95% confidence interval |
| Gender (male) | 51 to 55 years | 0.00167 | 0.00239 | 0.70 | 0.484 | [-0.0030;0.0064] |
|  | 56 to 60 years | 0.00166 | 0.00238 | 0.70 | 0.487 | [-0.0030;0.0063] |
|  | 61 to 65 years | 0.00167 | 0.00238 | 0.70 | 0.482 | [-0.0030;0.0063] |
|  | 66 to 70 years | 0.00169 | 0.00238 | 0.71 | 0.478 | [-0.0030;0.0063] |
|  | 71 to 75 years | 0.00168 | 0.00237 | 0.71 | 0.478 | [-0.0030;0.0063] |
|  | 76 to 80 years | 0.00167 | 0.00237 | 0.71 | 0.480 | [-0.0030;0.0063] |
|  | 81 to 85 years | 0.00165 | 0.00236 | 0.70 | 0.486 | [-0.0030;0.0063] |
|  | 86 to 90 years | 0.00165 | 0.00236 | 0.70 | 0.485 | [-0.0030;0.0063] |
|  | 91 to 95 years | 0.00157 | 0.00236 | 0.67 | 0.505 | [-0.0031;0.0062] |
|  | 95+ years | 0.00148 | 0.00235 | 0.63 | 0.530 | [-0.0031;0.0061] |
| Gender (female) | 51 to 55 years | 0.00164 | 0.00239 | 0.69 | 0.493 | [-0.0030;0.0063] |
|  | 56 to 60 years | 0.00166 | 0.00238 | 0.70 | 0.487 | [-0.0030;0.0063] |
|  | 61 to 65 years | 0.00167 | 0.00238 | 0.69 | 0.488 | [-0.0030;0.0063] |
|  | 66 to 70 years | 0.00167 | 0.00238 | 0.70 | 0.484 | [-0.0030;0.0063] |
|  | 71 to 75 years | 0.00166 | 0.00237 | 0.70 | 0.486 | [-0.0030;0.0063] |
|  | 76 to 80 years | 0.00163 | 0.00237 | 0.69 | 0.491 | [-0.0030;0.0063] |
|  | 81 to 85 years | 0.00161 | 0.00237 | 0.68 | 0.496 | [-0.0030;0.0063] |
|  | 86 to 90 years | 0.00157 | 0.00236 | 0.66 | 0.506 | [-0.0031;0.0062] |
|  | 91 to 95 years | 0.00159 | 0.00236 | 0.67 | 0.500 | [-0.0030;0.0062] |
|  | 95+ years | 0.00141 | 0.00236 | 0.60 | 0.549 | [-0.0030;0.0060] |
| demographics |  | 7.26*10-8 | 1.58*10-7 | 0.46 | 0.646 | [-2.4*10^-7^;3.8*10^-7^] |
| constant |  | -2.5815 | 4.7419 | -0.54 | 0.586 | [-11.877;6.714] |

**Appendix 2: Estimating thresholds per patient category**

We estimate the following equations, including interactions with gender, age group and disease group (see tables 8-10):

$${\log(Q}_{i,t})=\alpha_{1}+{\alpha_{2}T_{i,t}+\beta}_{1}Log\left( C_{i,t} \right)*{gender}_{i,t}+{{\frac{1}{2}\beta}_{2}Log\left( C_{i,t} \right)}^{2}+\theta_{1}Log\left( N_{i,t} \right)+{\frac{1}{2}\theta_{2}Log\left( N_{i,t} \right)}^{2}+\beta_{3}Log\left( C_{i,t} \right)Log\left( N_{i,t} \right)+\varepsilon_{i}+\epsilon_{i,t}$$

$${\log(Q}_{i,t})=\alpha_{1}+{\alpha_{2}T_{i,t}+\beta}_{1}Log\left( C_{i,t} \right)*{age group}_{i,t}+{{\frac{1}{2}\beta}_{2}Log\left( C_{i,t} \right)}^{2}+\theta_{1}Log\left( N_{i,t} \right)+{\frac{1}{2}\theta_{2}Log\left( N_{i,t} \right)}^{2}+\beta_{3}Log\left( C_{i,t} \right)Log\left( N_{i,t} \right)+\varepsilon_{i}+\epsilon_{i,t}$$

$${\log(Q}_{i,t})=\alpha_{1}+{\alpha_{2}T_{i,t}+\beta}_{1}Log\left( C_{i,t} \right)*{ICD group}_{i,t}+{{\frac{1}{2}\beta}_{2}Log\left( C_{i,t} \right)}^{2}+\theta_{1}Log\left( N_{i,t} \right)+{\frac{1}{2}\theta_{2}Log\left( N_{i,t} \right)}^{2}+\beta_{3}Log\left( C_{i,t} \right)Log\left( N_{i,t} \right)+\varepsilon_{i}+\epsilon_{i,t}$$

| *Table 8: estimation results by gender* | | | | | | |
| --- | --- | --- | --- | --- | --- | --- |
| N=11,079 | F(7,11078) = 2313.07 | | | Prob>F=0.000 | | R^2^=0.9492 |
| *gender* | *coefficient* | *Robust standard error* | *p-value* | elasticity | Threshold value | Bootstrapped 95%-CI |
| Male | -0.03 | 0.08 | 0.673 | -0.145 | € 84,900 | € 58,000-€ 112,000 |
| Female | -0.06 | 0.08 | 0.46 | -0.167 | € 65,100 | € 43,000-€ 88,000 |
| Ln(spending)^2^ | 0.00 | 0.01 | 0.593 |  |  |  |
| Ln(spending)*Ln(treatments) | -0.04 | 0.01 | 0.004 |  |  |  |
| Ln(treatments) | 1.30 | 0.08 | 0 |  |  |  |
| Ln(treatments)^2^ | 0.02 | 0.01 | 0.003 |  |  |  |
| Time trend | -0.02 | 0.00 | 0 |  |  |  |
| Constant | 35.44 | 3.47 | 0 |  |  |  |

| *Table 9: estimation results by age group* | | | | | | |
| --- | --- | --- | --- | --- | --- | --- |
| N=11,079 | | F(26,11078) = 724.56 | | | Prob>F=0.000 | R^2^=0.9300 |
| *Age group* | *coefficient* | *Robust standard error* | *p-value* | elasticity | Threshold value | Bootstrapped 95%-CI |
| 0 years | -0.014 | 0.072 | 0.849 | -0.043 | € 418,000 | € -112,000,000 - € 111,000,000 |
| 1 to 5 years | -0.120 | 0.081 | 0.141 | -0.176 | € 60,400 | € 21,000 - € 100,000 |
| 6 to 10 years | -0.143 | 0.084 | 0.089 | -0.197 | € 47,300 | € 3,000 - € 91,000 |
| 11 to 15 years | -0.064 | 0.087 | 0.464 | -0.120 | € 83,000 | € -181,000 - € 347,000 |
| 16 to 20 years | -0.089 | 0.083 | 0.285 | -0.151 | € 67,100 | € 37,000 - € 97,000 |
| 21 to 25 years | -0.058 | 0.082 | 0.479 | -0.125 | € 85,000 | € 35,000 - € 135,000 |
| 26 to 30 years | -0.109 | 0.087 | 0.21 | -0.180 | € 66,400 | € -6,000 - € 138,000 |
| 31 to 35 years | -0.130 | 0.090 | 0.146 | -0.204 | € 59,400 | € 22,000 - € 97,000 |
| 36 to 40 years | -0.082 | 0.087 | 0.347 | -0.161 | € 73,300 | € -21,000 - € 168,000 |
| 41 to 45 years | -0.076 | 0.083 | 0.363 | -0.162 | € 72,500 | € -2,000 - € 147,000 |
| 46 to 50 years | -0.152 | 0.082 | 0.064 | -0.242 | € 48,500 | € 36,000 - € 61,000 |
| 51 to 55 years | -0.093 | 0.079 | 0.243 | -0.185 | € 63,200 | € 47,000 - € 80,000 |
| 56 to 60 years | -0.076 | 0.084 | 0.368 | -0.171 | € 68,400 | € 36,000 - € 100,000 |
| 61 to 65 years | -0.055 | 0.078 | 0.482 | -0.153 | € 76,800 | € 50,000 - € 104,000 |
| 66 to 70 years | -0.087 | 0.077 | 0.257 | -0.185 | € 64,300 | € 47,000 - € 82,000 |
| 71 to 75 years | -0.085 | 0.078 | 0.278 | -0.179 | € 66,000 | € 45,000 - € 88,000 |
| 76 to 80 years | -0.105 | 0.077 | 0.173 | -0.194 | € 59,100 | € 44,000 - € 75,000 |
| 81 to 85 years | -0.077 | 0.075 | 0.304 | -0.158 | € 66,600 | € 42,000 - € 91,000 |
| 86 to 90 years | -0.069 | 0.074 | 0.356 | -0.133 | € 68,000 | € 41,000 - € 96,000 |
| 91 to 95 years | -0.040 | 0.070 | 0.57 | -0.075 | € 115,400 | € 48,000 - € 183,000 |
| 95+ years | -0.051 | 0.073 | 0.483 | -0.048 | € 193,400 | € 76,000 - € 310,000 |
| Ln(spending)^2^ | 0.005 | 0.006 | 0.363 |  |  |  |
| Ln(spending)* Ln(treatments) | -0.038 | 0.012 | 0.001 |  |  |  |
| Ln(treatments) | 1.316 | 0.077 | 0.000 |  |  |  |
| Ln(treatments)^2^ | 0.022 | 0.007 | 0.002 |  |  |  |
| Time trend | -0.018 | 0.002 | 0.000 |  |  |  |
| Constant | 35.124 | 3.348 | 0.000 |  |  |  |

| *Table 10: estimation results by disease group* | | | | | | |
| --- | --- | --- | --- | --- | --- | --- |
| N=11,079 | F(22,11078) = 1393.34 | | | Prob>F=0.000 | | R^2^=0.8688 |
| *Disease group* | *coefficient* | *Robust SE* | *p-value* | elasticity | Threshold value | Bootstrapped 95%-CI |
| Infectious diseases | -0.08 | 0.08 | 0.279 | -0.162 | € 53,500 | € 32,000 - € 75,000 |
| Neoplasms | -0.07 | 0.07 | 0.311 | -0.148 | € 68,300 | € 28,000 - € 109,000 |
| Diseases of the blood | -0.01 | 0.08 | 0.89 | -0.086 | € 201,800 | € -362,000 - € 766,000 |
| Endocrine diseases | -0.05 | 0.08 | 0.51 | -0.133 | € 75,700 | € 36,000 - € 116,000 |
| Mental disorders | -0.04 | 0.07 | 0.585 | -0.106 | € 64,000 | € 41,000 - € 87,000 |
| Diseases of the nervous system | -0.23 | 0.10 | 0.019 | -0.340 | € 33,500 | € 14,000 - € 53,000 |
| Diseases of the eye and ear | 0.01 | 0.07 | 0.835 | -0.125 | € 42,700 | € 28,000 - € 57,000 |
| Diseases of the circulatory system | -0.05 | 0.08 | 0.481 | -0.141 | € 95,700 | € 30,000 - € 161,000 |
| Diseases of the respiratory system | -0.07 | 0.08 | 0.407 | -0.174 | € 59,600 | € 28,000 - € 92,000 |
| Diseases of the digestive system | -0.05 | 0.08 | 0.562 | -0.151 | € 116,800 | € 82,000 - € 151,000 |
| Diseases of the skin | -0.03 | 0.07 | 0.7 | -0.154 | € 38,500 | € 20,000 - € 57,000 |
| Diseases of the musculoskeletal system | -0.04 | 0.07 | 0.626 | -0.143 | € 117,200 | € 86,000 - € 148,000 |
| Diseases of the genitourinary system | -0.03 | 0.07 | 0.657 | -0.121 | € 138,400 | € 100,000 - € 177,000 |
| Pregnancy, childbirth and perinatal period | -0.03 | 0.08 | 0.674 | -0.099 | € 215,800 | € 120,000 - € 312,000 |
| Congenital abnormalities | -0.06 | 0.08 | 0.463 | -0.106 | € 102,800 | € 68,000 - € 137,000 |
| Symptoms not elsewhere classified | -0.01 | 0.08 | 0.864 | -0.112 | € 71,700 | € 50,000 - € 94,000 |
| External causes | 0.16 | 0.08 | 0.054 | -0.093 | € 129,300 | € -1,170,000 - € 1,429,000 |
| Ln(spending)^2^ | 0.00 | 0.01 | 0.516 |  |  |  |
| Ln(spending)*Ln(treatments) | 0.02 | 0.01 | 0.009 |  |  |  |
| Ln(treatments) | 1.29 | 0.08 | 0.000 |  |  |  |
| Ln(treatments)^2^ | -0.04 | 0.01 | 0.004 |  |  |  |
| Time trend | -0.02 | 0.00 | 0.000 |  |  |  |
| Constant | 33.68 | 3.42 | 0.000 |  |  |  |

**Appendix 3: Robustness checks**

Table 11 summarizes the results of the robustness checks. Individual regression outcomes, specifics and descriptive statistics are available from the authors on demand.

| Table 11: summary of robustness checks | | | | |
| --- | --- | --- | --- | --- |
|  | Robustness checks | **Description** | **Estimated threshold** | **Confidence intervals** |
| 1 | Discount rate | Discount rate is varied between 0% and 5%. | € 73,600 per QALY (1,5%) | € 66,500 (0%) to € 90,200 (5%) per QALY |
| 2 | Cobb-Douglas specification | A specific health production function with diminishing marginal returns and constant elasticity of substitution. | €140,000 per QALY | € 113,000 - € 185,000 per QALY |
| 3 | Cobb-Douglas specification, mortality only | Cobb-Douglas health production function, only patient groups with mortality analyzed | €104,000 per QALY | € 88,000 - € 129,000 per QALY |
| 4 | Linear specification | A linear production function with constant marginal returns and elasticity of substitution | €184,000 per QALY | € 115,000 - € 472,000 per QALY |
| 5 | Random effects specification | Translog production function allowing for random variation in the estimated effect | Not valid | Hausman test rejected (P<0.0001) |
| 6 | Proportional multimorbidity correction | Patient groups are defined on first opened DRG, mortality is appointed proportionally | €201,000 per QALY | € 143,000 - € 271,000 per QALY |
| 7 | Estimated multimorbidity correction | Patient groups are defined on first opened DRG, mortality is estimated on spending patterns | €49,600 per QALY | € 46,000 - € 63,000 per QALY |
| 8 | Per patient specification | Translog model relating per patient outcomes to per patient spending | €176,000 per QALY | € 116,000 - € 237,000 per QALY |
| 9 | Linear per patient specification | Linear model relating per patient outcomes to per patient spending | €85.000 per QALY | € 58,000 - € 153,000 per QALY |
| 10 | Only mortality as outcome | Only mortality QALYS are used as oucome measure in the translog function | €89.000 per QALY | € 75,000 - € 102,000 per QALY |
| 11 | Estimation of cost per death avoided | Mortality is used as outcome measure in the translog model | € 475,000 per life lost (61-65 years) | €12 million (0 years) – €43,000 (96+ years) |
| 12 | Estimation of cost per death avoided is transformed to QALYs | Elasticity of spending per death avoided is used as input to calculate threshold of euro per QALYs | €44,000 per QALY (61-65 years) | €318,000 (0 years) - €33,000 (96+ years) per QALY |
| 13 | One year lag of the outcome measure | Spending in year t influences QALYs lost in year t+1 | Negative marginal effects | incalculable |
| 14 | Two year outcome measure | Spending in year t influences QALYs lost in year t plus in year t+1 | €60,000 per QALY | € 41,000 - € 79,000 per QALY |
| 15 | Adjusting for technology shocks | Year dummies are included in the baseline model | € 73,600 per QALY | € 59,000 - € 88,000 per QALY |
| 16 | Health trends are added | Obesity, smoking and heavy alcohol use are added as health trends | € 73,600 per QALY | € 59,000 - € 88,000 per QALY |

1. Discount rate

Benefits experienced in the far future are, according to economic theory, less valuable than the same benefits experienced immediately. The same would hold for QALYs averted: the first year additional lived in good health is valued more than the last additional year lived in good health. Standard Dutch guidelines recommend discounting nonmonetary benefits by 1.5%, but other discount rates have been used in the literature (e.g. Claxton et al., 2015 and Cutler et al., 2006 use a zero discount rate, while Hall and Jones, 2004 use a discount rate up to 6%). A higher discount rate reduces the value of QALYs in the far future, which disproportionately affects deaths averted in low age groups. Consequentially, thresholds increase when high discount rates are used.

2. Cobb-Douglas specification

The Cobb-Douglas (CD) function is often used to model a health production function displaying diminishing marginal returns. The CD-model has less flexibility than a translog model, i.e. a translog model is a generalization of the CD model. More specifically, the CD-model assumes constant returns to scale and constant elasticity of substitution, which both are unlikely to hold in diverse patient groups. For example, in small patient groups substitution between spending more per patient or helping more patients may be limited (non-constant elasticity of substitution), while in large patient groups the effect of additional spending may be lower than in small patient groups (non-constant returns to scale). These factors appear to have a large impact: the estimated threshold is significantly higher than in the preferred model.

3. Cobb-Douglas specification including patient groups with positive mortality only

Benefits may be measured less precisely in patient groups without mortality, for example, in the case of pregnancy, benefits of additional spending may be understated. As a robustness check, the CD-model is run on a subsample of patient groups with positive mortality. The estimated threshold is lower than in the full CD-model, indicating that benefits may be understated in the patient groups without mortality.

4. Linear specification

Linear models have been used in literature, and one advantage could be that the estimates are easy to interpret economically: one additional euro increases QALYs by β. However, since spending is distributed non-normally, log-transformations are preferred. The linear model renders a rather high threshold, signalling that estimated benefits may be lower in the high-end of the spending distribution.

5. Random effects specification

The preferred translog model is estimated using a fixed-effects linear regression, as is usual in time-series estimation (e.g. Edney et al., 2018). However, under the condition that the dependent variables are uncorrelated to the error terms, a random-effects model may be more efficient. To test this condition of uncorrelated error terms, a Hausman test is performed. Given that endogeneity of the fixed patient group characteristics may be expected in this analysis, it is unsurprising that the Hausman test is rejected. This implies that the random-effects model produces invalid estimates, and verifies that the fixed-effects model is preferred.

6. Proportional multimorbidity correction

In the main analysis, crossover effects are not allowed, i.e. spending on diabetes by definition does not affect mortality of diseases other than diabetes. A multimorbidity correction could relax this assumption. To this aim, spending on all morbidities is aggregated per patient, and “unique patient groups” are composed based on the first diagnosis in that year. For example, for male patients 70-75 for which the first diagnosis in a given year is CVA, all subsequent costs on CVA and all other diagnoses are aggregated. Next, a number of deaths need to be assigned to that specific patient group, consisting in part of the number of deaths on CVA and in part of the number of deaths on other secondary diseases. One could assume that the number of deaths is proportional to the spending on each disease category in that group. E.g. if 40% of spending on CVA is taken place in that patient group, 40% of deaths on CVA are appointed to that patient group. Furthermore, if 10% of spending on cancer takes place in the patient group with primary disease CVA, then 10% of deaths are appointed to that unique patient group of CVA patients. This would allow estimating the effect of increases in spending on the unique patient group other than on the primary diagnosis. A downside of the proportional multimorbidity correction is that spending increases per definition increase the number of deaths appointed to that patient group. This creates a positive correlation between spending and outcomes, which is reflected by the relatively high estimated threshold of about €200,000 per QALY.

7. Estimated multimorbidity correction

To correct for the bias of the proportional multimorbidity correction, the assumption that deaths are proportional to spending is relaxed. To this aim, the relation between deaths and spending is estimated for each of the 405 disease groups, after which the predicted deaths per unique patient group are used as input for the model in figure 1. However, due to the non-negativity restriction for the number of deaths, censoring bias at zero is expected to lead to overestimation of the effect, and a threshold biased downwards. The estimated threshold of €49,500 per QALY confirms this. While multimorbidity corrections may potentially improve the accuracy of the estimation, the current approaches may be more prone to bias than our preferred specification. However, this is a promising area of future research.

8. Per-patient specification

Expressing spending and mortality per patient has been used before in the literature (e.g. Van Baal et al., 2018). In our framework, a per-patient specification is less appropriate, as changes in spending per patient may result from changes in spending or changes in the number of patients. The latter induces the risk of omitted variable bias, as positive health trends may reduce both per-patient spending and health outcomes. The preferred translog model corrects for OVB by separating the effect of spending and the effect of changes in patients on outcomes, by assuming that health trends are mainly reflected in changes in patient numbers (see main text). Moreover, the per-patient model is less flexible in modelling the relation between spending, the number of patients and health outcomes over diverse patient groups than the translog model. The risk of OVB is reflected in the relatively high estimated threshold of €176,000 per QALY.

9. Linear per-patient specification

The linear per-patient specification is the least flexible model to relate health spending to outcomes, as it combines the disadvantages of the previous specification with high sensitivity to outliers due to the skewed distribution of health spending. Still, this model produces statistically significant outcomes (€85,000 per QALY), suggesting that the functional form of the model may affect the size of the threshold, but does not drive the relation between hospital spending and health outcomes, as all functional forms exhibit statistically significant results in the expected direction.

10. Only mortality as outcome

Removing step 1.1 to 1.4 in figure 1 from the estimation methodology renders a threshold estimation of €89,000 per QALY. This estimation may not take into account potential gains in non-mortality related quality of life, biasing the estimator upwards.

11. Estimation of costs per death avoided

Directly estimating the effect of changes in spending on mortality per age category results in estimations ranging from €12 million per death avoided for age 0 to €43.000 per death avoided for age 95+. This is highly consistent with estimations from Felder (2006) and Hall & Jones (2004).

12. Transformation of estimation of cost per death avoided to QALYs

Transforming the cost per death avoided to QALYs, using remaining healthy life expectancy and mean burden-of-disease estimates, renders QALY thresholds between €318,000 Per QALY (0 years) to €33,000 per QALY (96+ years). The mean age-group weighed threshold of €44,000 per QALY is roughly comparable the mortality-only estimation of the main model (€61,100 euro per QALY). As in 10, the estimation does not take into account potential gains in non-mortality related quality of life. Furthermore, the assumption that costs to save a life differ between age and gender categories requires estimation of age- and gender specific parameters. Moreover, stringent assumptions are necessary to extrapolate the findings to patient groups without mortality (Barnsley et al., 2013). Therefore, the preferred specification makes better use of available data.

13. One year lag of the outcome measure

Relating spending in year t to outcomes in year t+1 renders insignificant, positive estimates, suggesting spending in year t increases mortality in year t+1. It is conceivable that if mortality in year t is reduced, the risk of mortality in year t+1 is increased. However, t the absence of spending data as cohorts (e.g. spending in year t for age group 61 to 65 and spending in year t-1 for age group 60-64) renders estimation of lagged spending effects in our dataset invalid. Furthermore, to estimate time-dependent effects robustly, a larger time-span than three years is warranted. Lastly, a lagged effect requires additional correction for spending in the last years of life (Howdon & Rice, 2018).

14. Two-year outcome measure

One potential solution to estimating the effect of spending both in the same year and in the next year is to construct 2-year QALY outcomes. This resulted in a slightly lower threshold estimate of €60,000 per QALY, suggesting that part of the benefits of additional spending accrue in the next year.

15. Adjusting for technology shocks

In the main model, a year trend corrects for spurious correlations in the data. To correct for spurious health shocks, time dummies were included in the translog model. This did not alter the outcomes, demonstrating that the results are not driven by time-dependent technology shocks.

16. Health trends are added

Adding health-related trends, e.g. percentage of smokers, alcohol use or obesity does not alter the results, providing further evidence that the results are not driven by confounding health trends, and that the number of patients in a patient group may be a good proxy for changes in health status of patient groups.
